# Six-minute walk distance after coronary artery bypass grafting compared with medical therapy in ischaemic cardiomyopathy

**DOI:** 10.1136/openhrt-2017-000752

**Published:** 2018-02-20

**Authors:** Ralph A H Stewart, Dominika Szalewska, Amanda Stebbins, Hussein R Al-Khalidi, John G H Cleland, Andrzej Rynkiewicz, Mark H Drazner, Harvey D White, Daniel B Mark, Ambuj Roy, Dragana Kosevic, Miroslaw Rajda, Marek Jasinski, Chua Yeow Leng, Wiwun Tungsubutra, Patrice Desvigne-Nickens, Eric J Velazquez, Mark C Petrie

**Affiliations:** 1 Green Lane Cardiovascular Service, Auckland City Hospital and University of Auckland, Auckland, New Zealand; 2 Rehabilitation Medicine, Medical University of Gdansk, Gdansk, Poland; 3 Department of Biostatistics and Bioinformatics (HRA), Duke Clinical Research Institute, Duke University School of Medicine, Durham, North Carolina, USA; 4 Division of Cardiology, Departments of Medicine, Duke Clinical Research Institute, Duke University School of Medicine, Durham, North Carolina, USA; 5 National Heart and Lung Institute, Royal Brompton and Harefield Hospitals, Imperial College, London, UK; 6 Department of Cardiology and Cardiosurgery, University of Warmia and Mazury, Olsztyn, Poland; 7 Department of Cardiology, University of Texas Southwestern Medical Center, Dallas, Texas, USA; 8 Department of Cardiology, All India Institute of Medical Sciences, New Delhi, India; 9 Department of Cardiology, Dedinje Cardiovascular Institute, Belgrade, Serbia; 10 Nova Scotia Health Authority, Queen Elizabeth II Health Science Centre, Halifax, Canada; 11 Department of Cardiac Surgery, Wroclaw Medical University, Wroclaw, Poland; 12 Department of Cardiology, National Heart Centre Singapore, Singapore; 13 Siriraj Hospital, Mahidol University, Bangkok, Thailand; 14 National Institutes of Health/National Heart, Lung and Blood Institute, Bethesda, Maryland, USA; 15 Department of Cardiology, Golden Jubilee National Hospital, Glasgow, UK

**Keywords:** coronary artery bypass grafting, ischemic cardiomyopathy, exercise capacity, six-minute walk distance, clinical trial

## Abstract

**Background:**

In patients with ischaemic left ventricular dysfunction, coronary artery bypass surgery (CABG) may decrease mortality, but it is not known whether CABG improves functional capacity.

**Objective:**

To determine whether CABG compared with medical therapy alone (MED) increases 6 min walk distance in patients with ischaemic left ventricular dysfunction and coronary artery disease amenable to revascularisation.

**Methods:**

The Surgical Treatment in Ischemic Heart disease trial randomised 1212 patients with ischaemic left ventricular dysfunction to CABG or MED. A 6 min walk distance test was performed both at baseline and at least one follow-up assessment at 4, 12, 24 and/or 36 months in 409 patients randomised to CABG and 466 to MED. Change in 6 min walk distance between baseline and follow-up were compared by treatment allocation.

**Results:**

6 min walk distance at baseline for CABG was mean 340±117 m and for MED 339±118 m. Change in walk distance from baseline was similar for CABG and MED groups at 4 months (mean +38 vs +28 m), 12 months (+47 vs +36 m), 24 months (+31 vs +34 m) and 36 months (−7 vs +7 m), P>0.10 for all. Change in walk distance between CABG and MED groups over all assessments was also similar after adjusting for covariates and imputation for missing values (+8 m, 95% CI −7 to 23 m, P=0.29). Results were consistent for subgroups defined by angina, New York Heart Association class ≥3, left ventricular ejection fraction, baseline walk distance and geographic region.

**Conclusion:**

In patients with ischaemic left ventricular dysfunction CABG compared with MED alone is known to reduce mortality but is unlikely to result in a clinically significant improvement in functional capacity.

**Trial registration number:**

NCT00023595.

**Key messages**

**What is already known about this subject?**

In previous clinical trials, functional capacity did not improve in patients with stable coronary artery disease after coronary revascularisation compared with medical therapy alone. However, no trials have evaluated effects of coronary artery bypass surgery (CABG) on an objective test of exercise capacity in patients with ischaemic left ventricular dysfunction.

**What does this study add?**

In this analysis from the Surgical Treatment in Ischemic Heart disease trial, 6 min walk distance was similar during 3-year follow-up for patients randomised to CABG and to medical therapy alone.

**How might this impact on clinical practice?**

In patients with ischaemic left ventricular dysfunction, CABG may be indicated to improve survival but is unlikely to improve functional capacity.

## Introduction

In patients with coronary heart disease and heart failure, exercise capacity is an important determinant of quality of life and a powerful predictor of mortality.^1^ Coronary revascularisation has the potential to improve exercise capacity by relieving ischaemic symptoms during exercise or decreasing cardiac dysfunction related to myocardial ischemia. However, previous trials have not demonstrated a clear improvement in functional capacity after coronary artery revascularisation compared with medical therapy (MED) alone. In the COURAGE trial, the largest randomised trial comparing percutaneous coronary intervention (PCI) with MED in patients with stable coronary heart disease, the PCI strategy resulted in a modest improvement in angina during the first 12 months, but no improvement in physical function assessed by questionnaire at or after 12 months.^2^ Smaller randomised trials have also not demonstrated sustained improvement in functional capacity with coronary revascularisation in patients with stable coronary artery disease.^3^ In the more recent ORBITA trial, there was no difference in angina or functional capacity in patients with angina and single vessel coronary artery disease who were randomised to PCI or a sham procedure.^4^

The Surgical Treatment for Ischemic Heart Failure (STICH) trial is the only large randomised trial to compare coronary artery bypass surgery (CABG) with optimal MED in patients with severe left ventricular (LV) dysfunction who have coronary artery disease amenable to revascularisation.^5^ During a median follow-up of ~10 years, patients randomised to CABG had lower all-cause and cardiovascular mortality compared with those randomised to optimal MED.^6^ Compared with patients with normal LV function, functional capacity is more likely to be limited in those with ischaemic LV dysfunction, and these patients could also have a greater potential to improve with revascularisation. We previously reported that baseline 6 min walk (6 MW) distance predicted mortality during follow-up in the STICH trial.^7^ The aim of this analysis was to determine whether CABG compared with MED alone increases 6 MW distance during follow-up in STICH trial participants.

## Methods

### Patient population

The STICH trial randomised 1212 patients with an LV ejection fraction of 35% or less and coronary artery disease suitable for revascularisation to either CABG and optimal MED or to MED alone.^5 6^ Optimal MED included ACE inhibitor and/or angiotensin receptor blocker, a β-blocker, an aldosterone antagonist and antiplatelet agents adjusted to optimal doses. Statins, diuretics and digitalis were individualised to patient-specific indications. The use of implantable defibrillators was encouraged. Patients were enrolled at 99 clinical sites in 22 countries between July 2002 and May 2007. The rationale, trial design and complete inclusion and exclusion criteria have been described previously.^8^ All patients provided written informed consent.

### Six-minute walk

**Figure 1:**
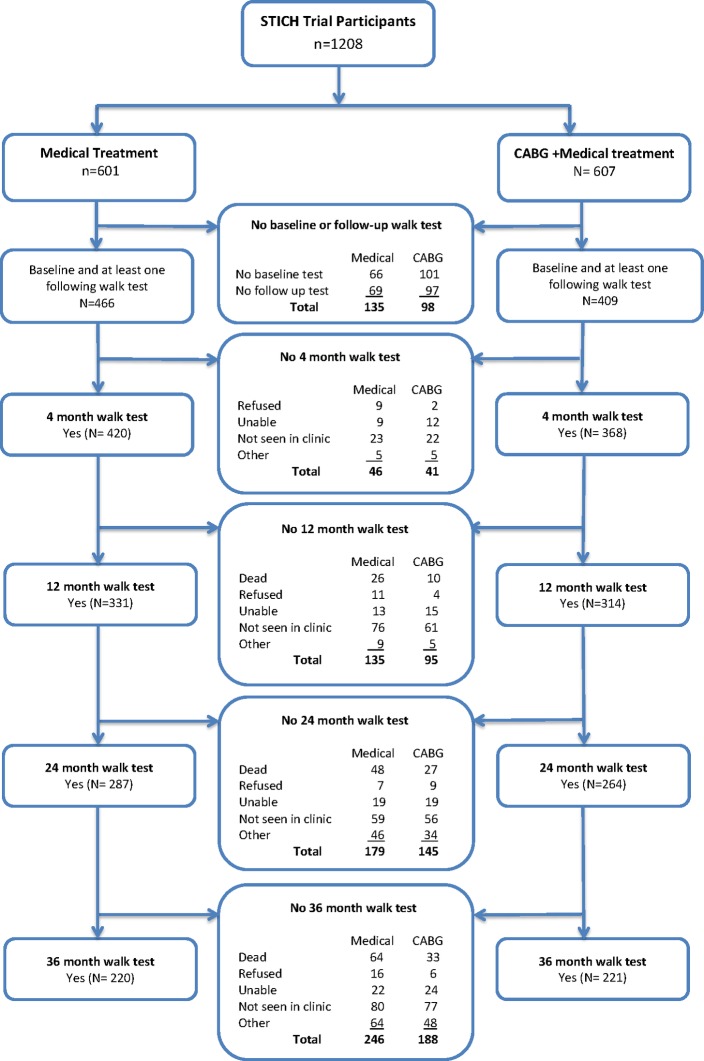
Summary of STICH trial patients included in the analysis of 6 min walk distance. Reasons for non-inclusion at each follow-up time are given. CABG, coronary artery bypass surgery; STICH, Surgical Treatment for Ischemic Heart Failure.

The trial protocol included a 6 MW test for all able study participants at baseline and the 4-month, 12-month, 24-month and 36-month follow-up assessments. The walk test was usually performed with distance marked in a long corridor free of obstacles. Instructions to patients included, ‘Walk for 6 min around this course, covering as much ground as possible during that time. Keep going continuously, if possible, but don’t worry if you have to slow down and rest’. Subjects were included in the current analysis if they completed both the baseline and at least one follow-up walk test (figure 1).

### Statistical analysis

All primary comparisons were performed with the treatment group defined according to the intention-to-treat principle. Descriptive statistics included counts (percentages) for categorical variables and medians (25th and 75th percentiles) or means with SD for continuous variables. The Pearson’s χ^2^ test was used for categorical variable comparisons. Changes in 6 MW distance from baseline to each follow-up time (4, 12, 24 and 36 months) were reported both as mean±SD, and median and 25% and 75% centiles.

Treatment comparisons were also evaluated using a linear mixed-effects model to account for repeated measures within a patient. In PROC MIXED in SAS, V.9.4, the baseline, 4-month, 12-month, 24-month and 36-month measurements for a patient were fitted using maximum likelihood methods with unstructured covariance matrix.^9^ This analysis included imputations for missing values for patients who were hospitalised at that time point or had died. For hospitalised patients, the worst non-null walk distance collected across the study was assigned. For patients who died, a value of 0 was used. At each time point, estimated treatment differences, 95% CI and P values were obtained using the model estimates. All reported P values were two sided. No adjustment was made in significance levels for multiple comparisons.

The difference in walk distances between baseline and 12 months are reported for subgroups defined for the primary analysis of the Surgical Treatment for Ischemic Heart Failure Extension Study (STICHES) trial.^6^ Results are also reported for patients with a Kansas City Cardiomyopathy Questionnaire (KCCQ) score^10^ and physical activity score^7^ above compared with below the median for the trial population.

## Results

Eight hundred seventy-five STICH trial patients (72%) who completed the 6 MW test at baseline and at least one follow-up assessment were included in this analysis. For these subjects, clinical characteristics were similar for those randomised to CABG (409, 67%) and MED (466, 77%) (table 1). Reasons for non-performance of the walk test at baseline and during follow-up are displayed by treatment allocation in figure 1.

**Table 1:** Baseline characteristics for study participants randomised to CABG and optimal medical therapy (MED) and to optimal MED

Baseline characteristics	All patients	MED	CABG	P value
Number of subjects	875	466	409	0.70
Age (years) Mean±SD	60.0±9.0	60.0±9.2	60.0±8.7	0.66
Male, n (%)	774 (88.5)	412 (88.4)	362 (88.5)	0.97
Region of enrolment, n (%)				0.80
Poland	273 (31.2)	145 (31.1)	128 (31.3)	
USA/Canada	151 (17.3)	83 (17.8)	68 (16.6)	
West Europe	73 (8.3)	42 (9.0)	31 (7.6)	
Other	378 (43.2)	196 (42.1)	182 (44.5)	
Ethnic minority, n (%)	270 (30.9)	138 (29.6)	132 (32.3)	0.40
Hyperlipidaemia, n (%)	526 (60.2)	285 (61.3)	241 (58.9)	0.48
Hypertension, n (%)	527 (60.2)	285 (61.2)	242 (59.2)	0.55
Diabetes, n (%)	344 (39.3)	189 (40.6)	155 (37.9)	0.42
Peripheral vascular disease, n (%)	117 (13.4)	70 (15.0)	47 (11.5)	0.13
Chronic renal insufficiency, n (%)	44 (5.0)	25 (5.4)	19 (4.7)	0.63
Atrial fibrillation/flutter, n (%)	105 (12.0)	59 (12.7)	46 (11.2)	0.52
Current smoker, n (%)	169 (19.3)	91 (19.6)	78 (19.1)	0.85
Depression, n (%)	42 (4.8)	20 (4.3)	22 (5.4)	0.45
Current angina class ≥II, n (%)	412 (72.7)	219 (73.7)	193 (71.5)	0.55
Current NYHA ≥III, n (%)	283 (32.3)	155 (33.3)	128 (31.3)	0.54
BMI (kg/m^2^)	27.4±4.6	27.4±4.7	27.4±4.5	0.85
LVEF (%)	28.3±8.9	28.6±9.1	27.9±8.7	0.41
LV end systolic volume index	85.7±36.3	87.1±37.5	84.1±34.9	0.30
Moderate-severe mitral regurgitation, n (%)	157 (18.0)	92 (19.8)	65 (15.9)	0.14
Three vessel/stenosis ≥75%, n (%)	304 (34.7)	156 (33.5)	148 (36.2)	0.40
Physical activity score*	71 (50.0, 88.0)	71 (50.0, 88.0)	71 (50.0, 88.0)	0.45
KCCQ score	64 (47.0, 80.0)	63.5 (47.0, 82.0)	64 (47.0, 79.0)	0.65

*Median (25th and 75th).

6 MW, 6 min walk; BMI, body mass index; CABG, coronary artery bypass grafting; KCCQ, Kansas City Cardiomyopathy Questionnaire; LV, left ventricular; LVEF, left ventricular ejection fraction; MR, mitral regurgitation; NYHA, New York Heart Association; STICH, Surgical Treatment for Ischemic Heart Failure trial.

**Figure 2:**
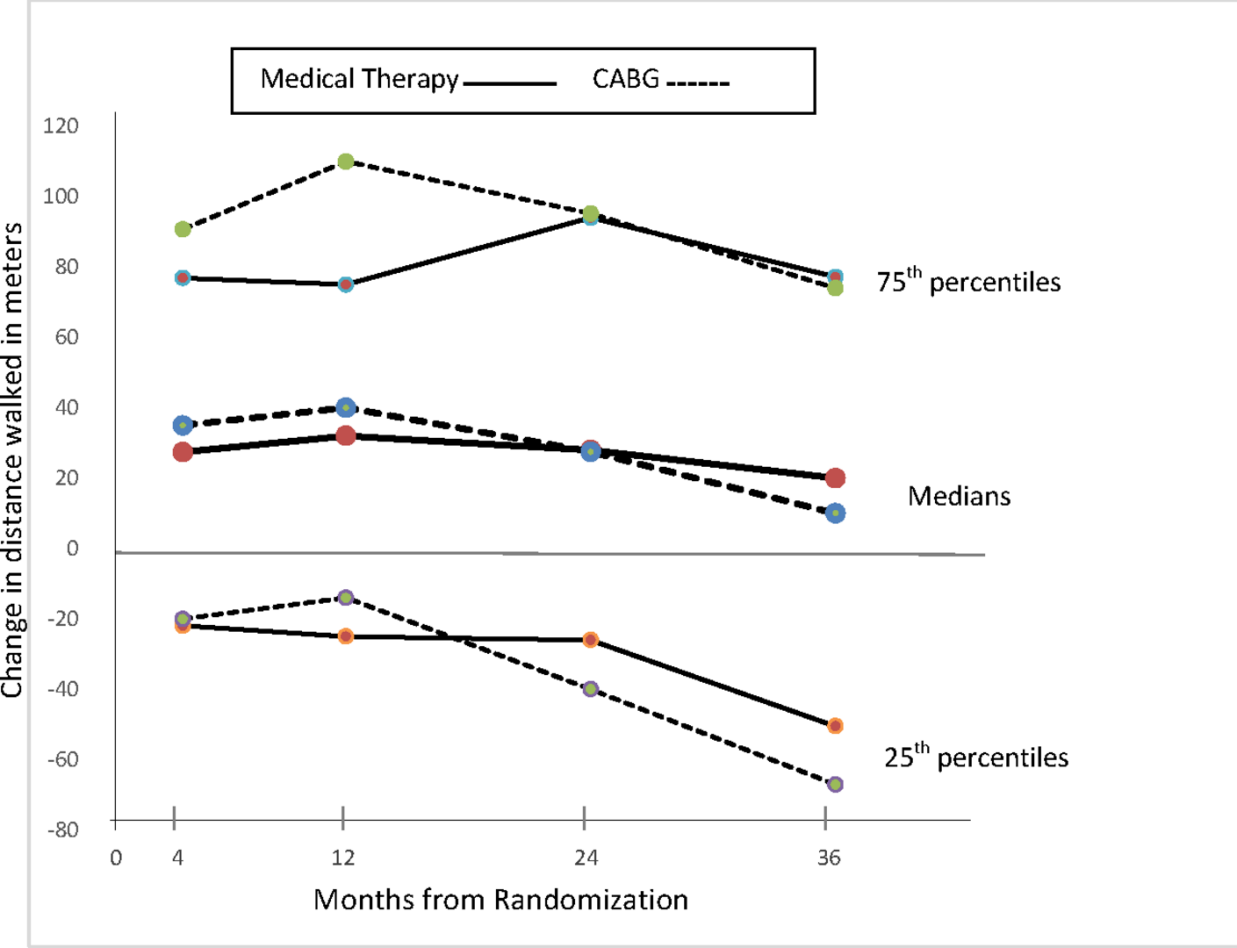
Change in 6 min walk distance from baseline to each follow-up time for subjects randomised to CABG and to medical therapy alone. The median change and 25th and 75th percentiles are displayed. Differences by treatment group were not statistically significant at any time. CABG, coronary artery bypass surgery.

6 MW distance was similar for subjects randomised to CABG compared with MED at baseline. For both groups, there was a modest increase in walk distance between baseline and 4, 12 and 24 months. Walk distance at 36 months was similar to baseline. The decrease in walk distance from 12 and 24 months to 36 months was greater for the 25th centiles compared with the medians and the 75th centiles, suggesting the temporal change reflected an increase in the number of subjects with a low walk distance (figure 2). The changes in walk distance between baseline and each follow-up assessment were similar by treatment allocation (table 2).

**Table 2:** Six-minute walk distance at baseline and change during follow-up for patients randomised to CABG compared with medical therapy only (MED)

	All patients	MED	CABG	P value
Baseline				
Number	875	466	409	
Mean±SD	340±117	339±116	340±117	0.79
Median (25th, 75th)	350 (274, 410)	348 (270, 410)	350 (276, 410)	
Change at 4 months				
Number	788	420	368	
Mean±SD	33±106	28±102	38±109	0.14
Median (25th, 75th)	30 (−20, 82)	27 (−22, 77)	35 (−20, 91)	
Change at 12 months				
Number	645	331	314	
Mean±SD	41±113	36±114	47±112	0.074
Median (25th, 75th)	35 (−20, 97)	32 (−25, 75)	40 (−14, 110)	
Change at 24 months				
Number	551	287	264	
Mean±SD	33±121	34±113	31±129	0.71
Median (25th, 75th)	28 (−32, 94)	28 (−26, 94)	27 (−40, 95)	
Change at 36 months				
Number	441	220	221	
Mean±SD	0±148	7±139	−7±157	0.48
Median (25th, 75th)	13 (−56, 75)	20 (−50, 77)	10 (−67, 74)	

Six-minute walk distance is in metres.

CABG, coronary artery bypass surgery.

In a repeated measures model that evaluated all visits, and with imputation for missing values, walk distance increased more for subjects randomised to CABG compared with MED only (difference +35 m, 95% CI 20 m to 51 m, P<0.001). After also adjustment for covariates (baseline distance walked, treatment received, age, LV end systolic volume index, history of stroke, creatinine, moderate severe MR and pulse rate) the change in walk distance averaged across all assessments was similar for CABG and MED groups, respectively (difference +8 m, 95% CI −7 m to 23 m, P=0.29).

**Figure 3:**
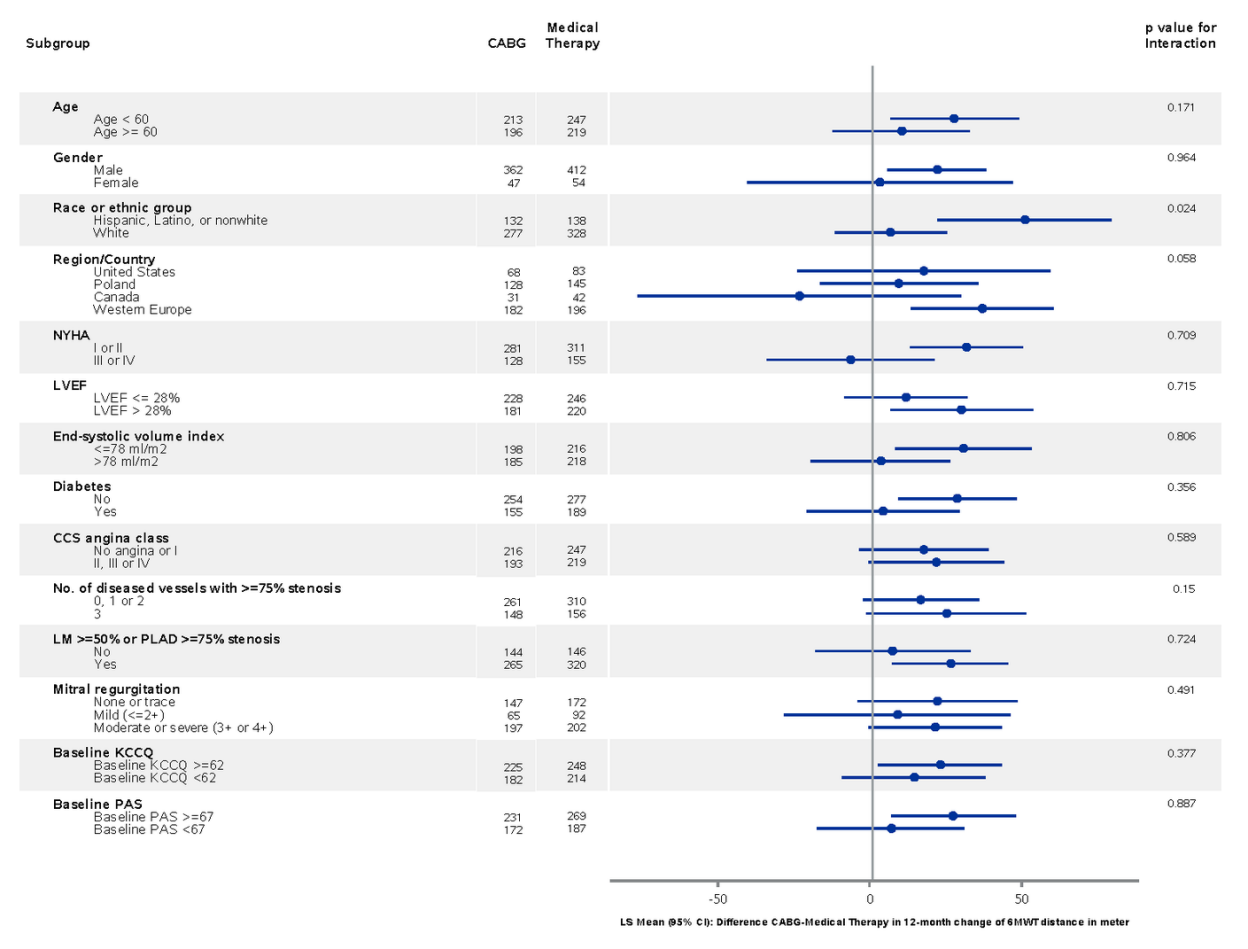
Change in 6 min walk distance at 12 months in subgroups defined by baseline characteristics. The mean (95% CI) for the difference between CABG and medical therapy groups for the change in 6 min walk distance between baseline and 12-month follow-up are presented. Walk distance is measured in metres. CABG, coronary artery bypass surgery; KCCQ, Kansas City Cardiomyopathy Questionnaire; LVEF, left ventricular ejection fraction; NYHA, New York Heart Association.

Change in walk distance at 12-month follow-up in subgroups are displayed in the forest plot (figure 3). There was a nominally significant interaction between race/ethnic group and increase in walk distance with CABG compared with MED alone. There were no other statistically significant interactions for difference in walk distance by treatment allocation for other baseline characteristics.

To determine whether change in 6 MW was prognostically important, the association between increases in 6 MW compared with baseline was determined for 10-year mortality. The HR for death for each 10 m increase in 6 MW between baseline and 4 months was 0.997 (95% CI 0.988 to 1.006), P=0.47, 12 months 0.984 (0.971 to 0.997), P=0.0018, 24 months 0.983 (0.973 to 0.994), P=0.002, and at 36 months 0.989 (0.981 to 0.998), P=0.022.

## Discussion

In this analysis from the STICH trial, there was no clinically important difference in 6 MW distance over 3 years for patients randomised to CABG compared with optimal MED alone. Trends for the 25th and 75th centiles by treatment group were similar to the median, suggesting ‘average’ changes were not masking a substantial improvement for some patients and worsening for others. Also subgroup analyses did not identify clinical features associated with a greater likelihood of improvement in walk distance with CABG. The difference in walk distance by treatment group were less than the minimally clinically evident change estimated from previous studies at ~25 m in coronary heart disease rehabilitation,^11^ ~44 m in pulmonary hypertension 12 and between 30 m and 50 m in heart failure trials.^13^

These observations are consistent with the absence of a clinically significant improvement in functional capacity after revascularisation, compared with MED only, reported in previous clinical trials of patients with stable coronary heart disease but without severe impairment of LV function.^2 3^ In the COURAGE trial, which randomised patients with stable CHD to either PCI or MED, there was a modest improvement in angina at 12 months, but not at 24 or 36 months, and no difference in functional capacity or quality of life by treatment allocation.^2^ Similar observations were reported from a meta-analyses that included additional smaller studies comparing coronary revascularisation to MED alone in patients with stable coronary heart disease.^3^

CABG could have a greater potential to improve exercise capacity in patients with angina, myocardial ischaemia or viable myocardium. However, STICH trial patients with angina compared with those without angina were not more likely to increase their 6 MW distance after CABG. Also previous subgroup analyses from STICH reported no difference in outcomes from CABG compared with MED by presence of angina,^14^ myocardial viability^15^ or myocardial ischaemia^16^ at baseline. Previous analyses from STICH have reported a small but statistically significant improvement in quality of life with CABG compared with MED^17^ and a modest decrease in angina (50% with CABG vs 41% with MED).^14^ A limitation of questionnaire-based assessment of quality of life, compared with more objective measures, is that responses may be influenced by knowledge of cardiac disease.^18^ In the ORBITA trial, which included a sham procedure, there was no significant improvement in exercise time or quality of life after PCI in patients with angina and single vessel coronary artery disease.^4^

We previously reported that baseline 6 MW distance predicted all-cause mortality after adjusting for other covariates.^7^ In the current analysis, we also report that both increase and decrease in 6 MW distance between baseline and 1 year are also associated with mortality risk. These observations suggest that functional capacity assessed by the 6 MW test is prognostically important in this study population. We also previously reported that patients who walked <300 m or had lower levels of physical activity assessed by questionnaire did not have a clear mortality benefit from CABG compared with those with greater exercise capacity.^7^ Knowledge that these patients also are unlikely to have an improvement in functional capacity from CABG compared with MED is important when considering the benefits of CABG in individual patients.

In the STICH trial, mortality was lower after a 10-year follow-up in patients randomised to CABG, with mortality benefit accruing gradually over time. At 1-year mortality was nominally higher after CABG.^6^ The small favourable effect of CABG on angina and quality of life reported previously were greatest at 1 year and less at 2-year and 3-year follow-up.^17^ In the current analysis, most data on 6 MW distance was available at 1 year, and assessments were only made to year 3. The results and timing of assessments in this study suggest the long-term survival benefit from CABG compared with MED in patients with ischaemic LV dysfunction are independent of changes in functional capacity.

### Study limitations

Baseline characteristics for patients included in the analysis were similar by treatment allocation, but fewer patients randomised to CABG completed the baseline walk test, and more patients randomised to MED compared with CABG had missing tests during follow-up, in part because of higher mortality. The reasons for baseline differences in completion of the 6 MW are uncertain. It is possible that imbalances in missing tests by treatment group resulted in a survival bias. This was evaluated in a sensitivity analysis with imputation of ‘0’ walk distance for patients who died, and the lowest reported walk distance for missing tests. Because slightly more patients in the MED group died during follow-up, these imputed values may underestimate 6 MW distance during follow-up for MED relatively more than the CABG group.

Walk distance is influenced by the motivation of the patient and the person supervising the test. These factors may have contributed to substantial variation in walk distance between assessments, and this decreases the value of the walk test for assessing change in functional capacity in individual patients. However, between tests, variation was similar for both randomised groups, and the 95% CIs exclude clinically important differences in average walk distance between treatment groups. During the first year of follow-up, ~10% of subjects randomised to MED cross over to CABG, which may act to decrease estimates of any benefits of CABG on functional capacity. The study was undertaken in many countries that may have different levels of medical or access to different therapies, but results were broadly similar across geographic regions. Randomisation and the intention-to-treat analysis would result in balancing of these and other unmeasured factors by treatment allocation.

The 6 MW test has been used for many years to evaluate treatment effects in clinical trials for heart failure, pulmonary hypertension and chronic respiratory disease. In randomised trials. 6 MW distance increased with cardiac resynchronisation therapy, consistent with benefits on other clinical outcomes and mortality.^13 19 20^ However, in studies of pharmacological therapies for heart failure, there has been no consistent correlation between change in 6 MW distance and clinical outcomes.^12 19^ In an analysis that included evaluation of 34 heart failure device or drug trials, there was a modest statistical correlation between the placebo corrected change in 6 MW distance and mortality.^20^ However, 6 MW distance improved in only 45% (10 of 22) of studies that evaluated therapies known to have a favourable effect on mortality.^20^ Meta-analyses of trials evaluating treatments for pulmonary hypertension have also reported no correlation between change in 6 MW distance and treatment effects on mortality.^12 19 21^ The current study suggests that even though CABG compared with MED reduces mortality, and higher exercise capacity is associated with lower mortality, CABG does not improve functional capacity in patients with ischaemic LV dysfunction.

## Conclusion

In patients with ischaemic LV dysfunction and coronary artery disease amenable to CABG, 6 MW distance was similar over 3-year follow-up after randomisation to CABG or to MED alone. Subgroup analysis did not identify patient characteristics associated with a greater increase in walk distance after CABG. These observations suggest that although CABG is known to improve survival over the longer term, it is unlikely to improve exercise capacity in most patients with ischaemic LV dysfunction.
